# Cloning, Sequencing and *In Silico* Analysis of Omp C of *Salmonella* Typhimurium

**DOI:** 10.5402/2012/512848

**Published:** 2012-03-05

**Authors:** Richa Jha, Anil Kumar, Anjani Saxena, Shantanu Tamuly, M. K. Saxena

**Affiliations:** ^1^Department of Molecular Biology and Genetic Engineering, G.B. Pant University of Agriculture & Technology, Pantnagar 263145, India; ^2^Animal Biotechnology Center, Department of Veterinary Physiology & Biochemistry, G.B. Pant University of Agriculture & Technology, Pantnagar 263145, India

## Abstract

*Salmonella* Typhimurium is an important pathogen having a broad host range. In human population it causes mostly gastroenteritis but there are reports in which it was found to be responsible to cause several lethal diseases like endocarditis and meningitis. Poultry products are the major sources of this organism in India as these are consumed at various stages of cooking. The available vaccines have their own limitations such as short-term immunity. Outer membrane proteins have shown some promising potential, so in the present study Omp C of *Salmonella* Typhimurium was cloned and sequenced to explore the possibility of development of r-DNA vaccine against *Salmonella* Typhimurium for poultry. The sequence of Omp C was studied for antigenic indexing, epitope mapping, and MHC mapping using various bioinformatic tools. The ORF analysis revealed a complete coding region of approximately 1000 bp. Five major and 13 minor B-cell epitopes were identified having an antigenic index of 1.7. The sequences also showed major histocompatibility complex (MHC) class I and class II binding region indicating a potential of eliciting cell-mediated immune response. The findings indicate that Omp C may be proven as promising candidate for development of r-DNA vaccine against *Salmonella* Typhimurium.

## 1. Introduction

Salmonellosis is a major foodborne illness and life-threatening problem worldwide caused by various serovars of *Salmonella* among which *Salmonella *Typhimurium is one of the most important serovars. In developing countries like India where the hygienic conditions are not very good salmonelloses have became major problem related to human health and poultry products are known to be significant reservoir for *Salmonella* and most important source to human infections [1, 2]. The available vaccines for poultry in India are not very effective [3, 4]. The backyard poultry practices are very common in India so there is always a possibility of transfering of *Salmonella *Typhimurium from poultry to human populations where it can cause serious health problems [5, 6]. There is an emergent need of better vaccine for poultry against salmonellosis.

 In last few years Omps have been studied and have shown their immunopotential [7–9]. Few of them have been characterized [10]. In earlier report Omp C has been studied [11], and it was found that Omp C was expressed during high salt concentration which was equivalent to human serum [12]. It was found be thermostable [13] and resistant for proteolysis [14]. This finding indicates that Omp C may be proven as an efficient candidate for vaccine development. As the isolation of individual Omp in large scale for development of an effective vaccine is a labour intensive and costly procedure, the present study deals with the cloning, sequencing, and *in silico* analysis of Omp C gene of *Salmonella *Typhimurium to explore the possibility of development of r-DNA vaccine against poultry salmonellosis.

## 2. Materials and Methods

### 2.1. Bacterial Strains

Culture of *Salmonella* Typhimurium (MTCC 3231) was procured from Institute of Microbial Technology, Chandigarh, India and maintained in Luria Bertini media. *Escherichia coli* DH5*α* used in cloning experiment was purchased from Bangalore Genei, India and grown in LB broth. 

Blunt cloning vector pJET 1.2, blunting enzyme, and T4 DNA ligase were procured from Qiagen, USA. The antibiotics (Ampicillin (100 *μ*g/mL) and Kanamycin (50 *μ*g/mL)) used for selection of recombinants were procured from Himedia, India.

The cultures were maintained in LB agar slants and their purity was tested by biochemical tests and *Salmonella *specific PCR.

### 2.2. Cloning of Omp C Gene

Genomic DNA was isolated by CTAB method [14]. Primers were designed for Omp C gene of *Salmonella *Typhimurium using sequence information available on NCBI and using gene tool software.

Forward primer—5′GGATCCATGCGTATCGGCTT3′.

Reverse primer—5′AAGCTTTTAGAACTGGTAAA3′. 

25 *μ*L PCR reaction mixture containing 40 ng of genomic DNA, 20 pmole of primers, 200 *μ*M of each dNTPs, 1.5 Mm MgCl_2_ and 2 U of jumpstart polymerase (Sigma, USA). The gene was amplified by PCR using the following programme, that is, initial denaturation at 94°C, followed by 30 cycles of denaturation at 94°C, annealing at 46°C, and elongation at 72°C. All the reagents used for PCR except enzyme were procured from Bangalore Genei, India. The amplified product was checked on 1% agarose gel and elution of the purified product was carried out using QIA quick gel extraction kit (Qiagen, USA). Blunting was carried out using standard kit procedures (pJET cloning kit, Qiagen, USA) and the product was cloned into pJET cloning vector through blunt end ligation. The ligate was transformed in chemically induced competent *E. coli* DH5*α* cells. Clones were inoculated in LB ampicillin tubes and plasmid was isolated by the alkaline lysis method and insert from plasmid was released by digestion with *Bam *HI and *Hind *III restriction enzymes.

The recombinant clones were screened to obtain insert of desired size verified by colony PCR amplification. The cloned product was sequenced by Ocimum Biosolutions Ltd., Hyderabad. The sequence was submitted to NCBI. 

### 2.3. Sequence Similarity and Phylogenetic Analysis

The sequence obtained was subjected to homology search using BLASTn (http://www.ncbi.nlm.nih.gov/). The sequence showing maximum similarity with Omp C was subjected to multiple sequence alignment by CLUSTALW and a phylogenetic tree was constructed, based on the comparative analysis of related protein sequences, using UPGMA method.

Amino acids sequence was translated using DNASTAR Inc, USA, software. Structural analysis of Omp C was carried out using different online servers, namely, Protparam (EXPA-syserver: Protparam) [7], Pfam (http://pfam.sanger.ac.uk/), and PDB server (http://www.ebi.ac.uk/pdbsum/).

## 3. Results and Discussion

The purity of the culture was checked by biochemical characterization and *Salmonella*-specific PCR. The culture was found to be MR+, VP-, Urease- biochemically, which is characteristic of *Salmonella* Typhimurium. In PCR amplification an amplicon of 496 bp was obtained which confirmed the identification through biochemical tests.

### 3.1. PCR Amplification and Cloning

**Figure 1:**
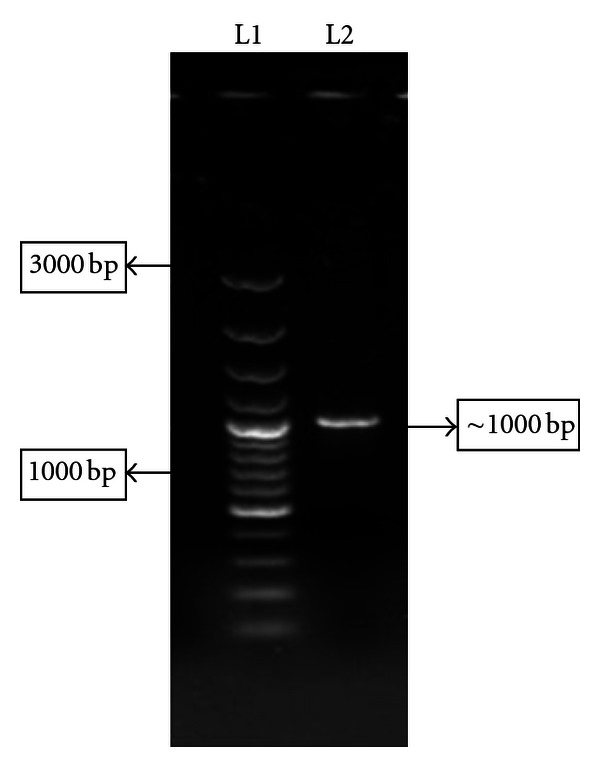
L1: GeneRuler 100 bp Plus DNA Ladder (MBI Fermentas). L2: PCR Product (~1000 bp).

The PCR amplification with Omp-C-specific primers was conducted with genomic DNA, which resulted in a product of approximate size 1000 bp (Figure 1). The desired product was successfully purified using QIA quick gel extraction kit and cloned in pJET 1.2 blunt cloning vector (Fermentas, USA) and transformed into chemically competent *E.coli *DH5*α* cells.

**Figure 2:**
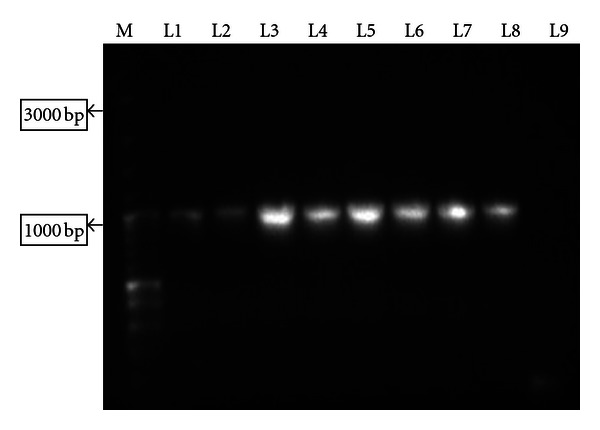
M: GeneRuler 100 bp Plus DNA Ladder (MBI Fermentas), L1–L8: PCR products of cloned plasmids, L9: negative control.

**Figure 3:**
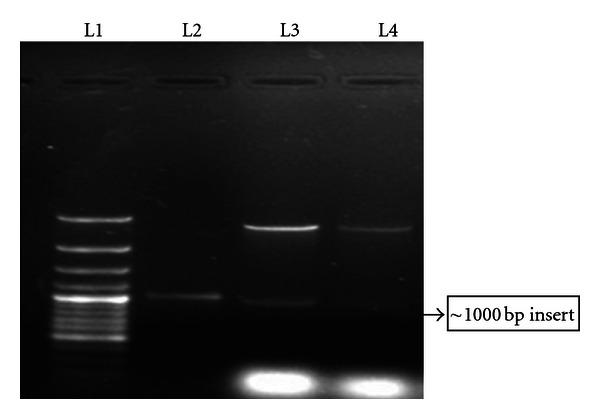
Release of insert through restriction digestion. L1: GeneRuler 100 bp Plus DNA Ladder (MBI-Fermentas), L2: eluted PCR product, L3-4: insert release form cloned recombinant plasmid.

Recombinant clones were selected by colony PCR (Figure 2). Restriction digestion of isolated recombinant plasmids was found to release an insert of ~1000 bp of Omp C gene (Figure 3). The insert was sequenced and complete cds was submitted in NCBI Genbank and assigned the *Accession no. JF896322*. The amplified product was found to be ~1 kb and the complete cds was of 993 bp having a GC content of 47.53%.

### 3.2. Sequence Analysis of *S*. Typhimurium Omp C Gene

**Figure 4:**
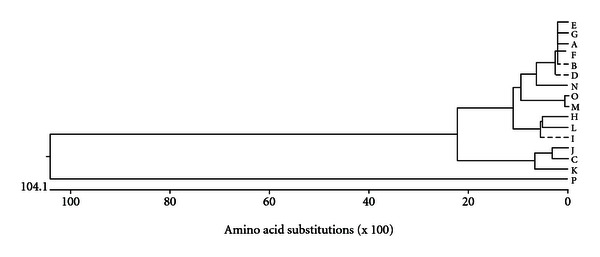
Phylogenetic tree of amino acid sequences of related sequences. A. Outer membrane protein N precursor [*Salmonella enterica *subsp. enterica serovar Wandsworth str. A4-580]. B. Hypothetical protein SPAB_01846 [*Salmonella enterica* subsp. enterica serovar Paratyphi B str. SPB7]. C. Porin, Gram-negative type [*Salmonella enterica *subsp. enterica serovar Dublin str. SD3246]. D. Outer membrane protein N [*Salmonella enterica *subsp. *enterica* serovar Heidelberg str. SL486]. E. Outer membrane protein N [*Salmonella enterica *subsp. enterica serovar Newport str. SL254]. F. Outer membrane protein [*Salmonella enterica *subsp. enterica serovar Gallinarum str. RKS5078]. G. Outer membrane protein N [*Salmonella enterica *subsp. enterica serovar Dublin str. CT_02021853]. H. Outer membrane protein [*Salmonella enterica *subsp. enterica serovar Montevideo str. LQC 10]. I. Outer membrane protein N precursor [*Salmonella enterica *subsp. enterica serovar Urbana str. R8-2977]. J. Outer membrane protein N precursor [*Salmonella enterica *subsp. enterica serovar Hvittingfoss str. A4-620]. K. Outer membrane protein N precursor [*Salmonella enterica *subsp. enterica serovar Mississippi str. A4-633]. L. Outer membrane protein N [*Salmonella enterica *subsp. enterica serovar Saintpaul str. SARA29]. M. Hypothetical protein STY1649 [*Salmonella enterica *subsp. enterica serovar Typhi str. CT18]. N. Outer membrane protein N [*Salmonella enterica *subsp. enterica serovar Javiana str. GA_MM04042433]. O. Outer membrane protein [*Salmonella enterica *subsp. enterica serovar Typhi str. E00-7866]. P. outer membrane protein C [*Salmonella enterica *subsp. enterica serovar Typhimurium].

The NCBI BLAST search of the Omp C gene showed maximum homology (99%) with outer membrane proteins of serovars like *S. enterica* serovar Typhi, Gallinarum, and Paratyphi. Multiple sequence alignment showed that it is closely related to Omp C of *S.* Typhi. The sequence shows 75% similarity with *E. coli *Omp C (Figure 4).

By bioinformatic analysis using DNA STAR software, the protein was found to have 330 amino acids. 24 amino acids were strongly basic (+) (K, R), 45 strongly acidic (−) (D, E), 95 hydrophobic (A, I, L, F, W, V), and 112 polar amino acids (N, C, Q, S, T, Y). It shows an isoelectric point at pH 4.02. At physiological pH the protein has a net negative charge (−21.08).

**Figure 5:**
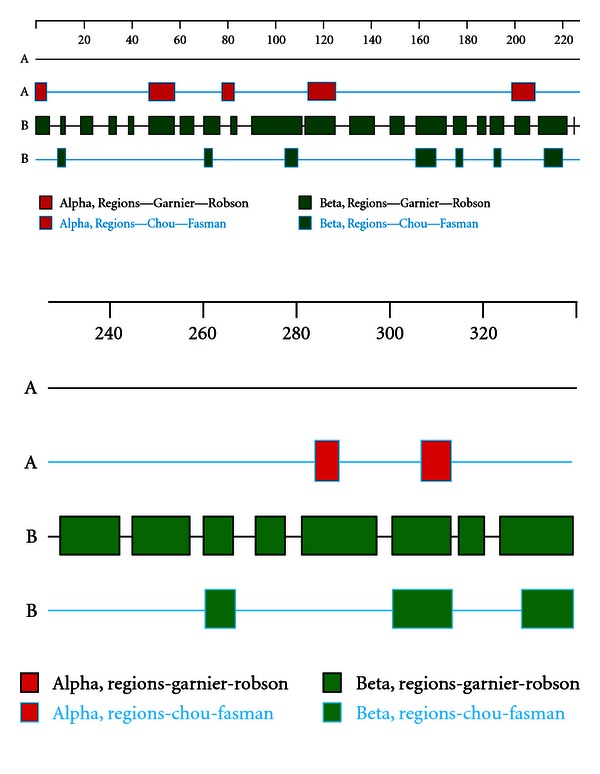
Structural analysis by DNASTAR software.

PSORTb v 3.0 analysis revealed the presence of noncytoplasmic signal peptide in the protein which indicated the subcellular localization of the protein. The location of the protein depends on the presence of signal peptide and membrane spanning alpha helixes. We found six major alpha helical regions throughout the protein by secondary structure analysis. There were more beta regions then alpha regions in Omp C gene (Figure 5).

Prosite analysis showed the presence of five motifs in Omp C gene, that is, protein kinase C phosphorylation site at four locations (10–12, 99–101, 107–109, 180–182), N-glycosylation site at four locations (38–41, 130–133, 241–244, 299–302), casein kinase II phosphorylation site at seven locations (40–43, 92–95, 134–137, 163–166, 178–181, 302–305, 324–327), N-myristoylation site at three locations (55–60, 168–173, 237–242), and tyrosine kinase phosphorylation site at two locations (145–153, 268–274).

Pfam analysis showed the protein to be a member of gram-negative porin family. Blastp was performed by Swissprot model analysis. This showed maximum similarity with Omp N of *Salmonella enterica* subsp. enterica serovars (99%). The protein sequence was also found to be 98% similar to outer membrane protein S2 of *Salmonella* Typhi and 96% to outer membrane protein N of *S. paratyphi* and *S. arizonae.*

**Figure 6:**
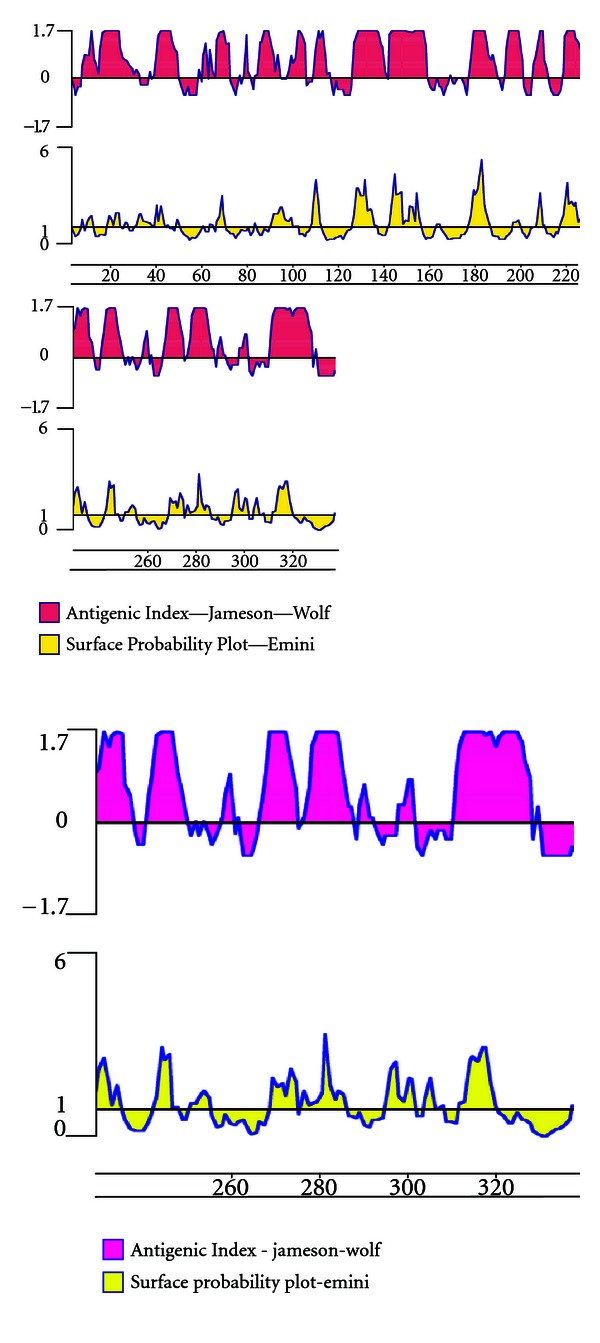
Antigenic index and Surface Probability plot of translated Omp C gene sequence.

Antigenic characterization of the translated protein sequence was done by protean [15]. The surface probability plot represented several hydrophilic exposed domains in the Omp C protein sequence. These exposed regions comprised the major epitopes of the protein, some of them being unique. The antigenic index collaborated with the hydrophilic regions and creates a linear surface contour profile of the protein. Antigenic sites were found to be located within the surface-exposed regions of the protein. Protean predicted five major epitopes, two of them occupying the 130–160 amino acid region and having maximum surface probability [16]. Approximately 13 minor epitopes were observed in the analysis. The epitopes have the maximum antigenic index of 1.7 (Figure 6).

#### 3.2.1. B-Cell Epitope Prediction

**Figure 7:**
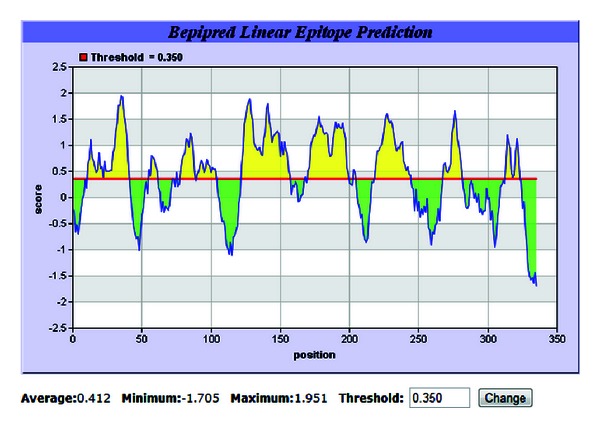
B-cell epitope prediction through IEDB online epitope prediction tool.

To find out the B-cell epitope regions of Omp protein the BepiPred prediction tool of IEDB analysis resources was used. The sequence was loaded into the tool window and searched for most potential linear epitopes (Figure 7).

Total of 13 B cell epitopes were analyzed (Table 1). Among these 13 epitopes mainly eight were found to be more immunogenic as predicted by the scores of epitope. A peptide length of 10 or more amino acid are supposed to be good B cell epitope.

#### 3.2.2. T-Cell Epitope Prediction

**Figure 8:**
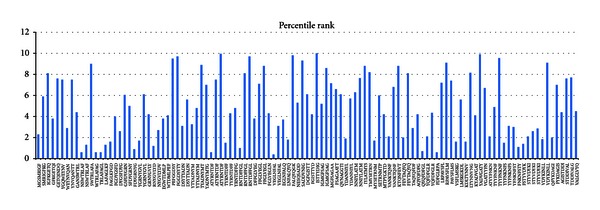
MHC I epitopes identified through IEDB epitope prediction tool.

A critical step in developing immune response against pathogens is the recognition of antigenic peptides presented by MHC class I and II molecules. Peptides are divided into binders and nonbinders and binding affinities of MHC class I and II are calculated by epitope database analysis and ranked according to their percentile. Total 14 MHC I epitopes were found in the analysis including 5 high affinity epitopes (Figure 8).

For MHC class II a percentile rank for each of the four methods (ARB, Combinatorial library SMM-align and Sturniolo) was generated by comparing the peptide score against the scores of five million 15 mers selected from SWISS-PROT database. A small percentile rank indicates high affinity. A graph was been plotted between percentile rank and amino acid position. There are three types of MHC class II reported in case of mice for example, H2 IAB, H2 IAD, H2 IED. Data has been analyzed by bar diagram against amino acid position and median percentile rank.

**Figure 9:**
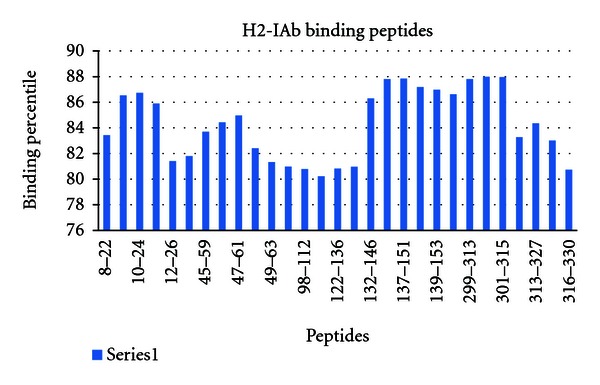
H2-IAb epitope prediction thorough IEDB.

**Figure 10:**
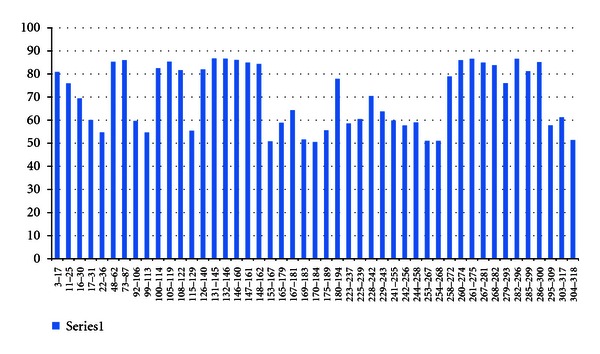
H2-IAd epitope prediction thorough IEDB.

**Figure 11:**
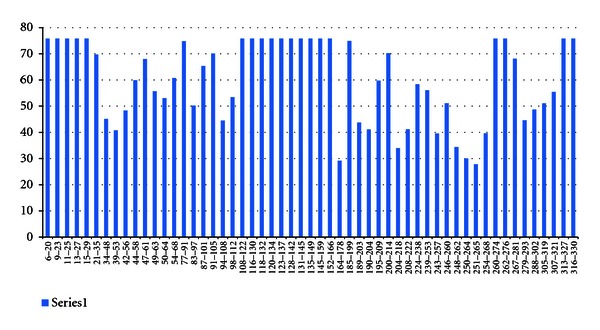
H2-IEd epitope prediction thorough IEDB.

In case of H2-IAb 12 high-affinity MHCII binding sites were found. In H2-IAd, 23 high-affinity MHCII binding sites were observed and in H2-IEd, and 19 high binding sites were found (Figures 9, 10, and 11).

It has been reported through multiple sequence alignment tools that *S*. Typhi Omp C consists of 8 variable regions on comparison with other porins with well-known crystal structures [17]. These variable regions have been found to be on the outer side of the membrane and therefore they have high probability to be presented for B-cell recognition and elicit immune response. These findings clearly depict that Omp C has recognized B-cell epitopes and as it shares maximum similarity with Omp C of *S*. Typhimurium (98%) these variable regions can be strongly predicted to act as possible B-cell epitopes capable of eliciting immune response.

Sequencing revealed that the C-terminus of Omp C has typical characteristic of Omps. The last residue at the C-terminus, phenylalanine, has been reported to be highly conserved among outer membrane proteins and is essential for stability and correct assembly of protein into the outer membrane [18]. The 15 C terminal amino acid residues of Omp C including the terminal phenylalanine were found to be hydrophobic in nature which is important for incorporation of the protein in the membrane.

Omp C of *Salmonella* has been purified using salt extraction procedures [19], and its epitopes have been mapped [20]. It is found to be a trimer made of 16 stranded *β*-barrel monomers and is a major cell surface antigen from the human pathogen *Salmonella typhi*. The assembly of trimer and the stability of the *β*-barrel have been found to be important for epitope presentation. The *Salmonella*-specific conformational epitope was found to be more stable than in case of *Enterobacteria *[20].

It is a good candidate to display heterologous epitopes on the cell surface [21, 22]. The functional and mature Omp C is a homotrimer. The monomer without the signal peptide has 357 amino acids and a molecular weight of 39 kDa. The purification and crystallization of native Ty21a Omp C have been described earlier [19]. Omp C is expressed not only under free living conditions, but also during infection, since the osmolarity of the human serum is equivalent to high salt conditions maintained in the laboratory [23]. These reasons suggest that Omp C could be a candidate antigen for diagnostics and vaccination. Omp C was found to be conserved within eleven *Salmonella *serotypes [11]. These findings indicate that Omp C can be in further studies for vaccine development against a range of serovars and its epitope mapping reveals its high immunogenic potential as an r-DNA vaccine candidate.

## Figures and Tables

**Table 1:** Major B-cell epitopes.

No.	Start Position	End Position	Peptide	Peptide length
1	11	40	ETQINDQLTGYGQWEYNVQANTTEGEGANS	30
2	55	61	GSFDYGR	7
3	72	73	WT	2
4	75	75	M	1
5	78	88	EFGGDSYTYAD	11
6	90	104	YMTGRANGVATYRNT	15
7	122	157	KNESQSADDVNIGTNNRNNGDDIRYDNGDGFGISTT	36
8	168	168	A	1
9	170	199	YTTSDRTNEQVNAGGTIAGGDKADAWTAGL	30
10	203	204	AN	2
11	218	244	MTPYGKTDAGYAGGVANKTQNFEVTAQ	27
12	268	281	NNVNGDDKDLVKYA	14
13	313	323	DAGISTDDIVA	11
